# Supplementary material for: Progress and stagnation: A comparative study of gender representation in Chinese language textbooks for junior high school between 2001 edition and 2023 edition
Source: PLoS One. 2025 Nov 11;20(11):e0334398. doi: 10.1371/journal.pone.0334398 (PMC12604804; doi:10.1371/journal.pone.0334398)
Supplement: Attachment — Submitted filename: Response to Reviewers(N).docx [file pone.0334398.s001.docx]

**Response to Reviewers**

1. *Please ensure that your manuscript meets PLOS ONE's style requirements, including those for file naming.*

**Response:** HAVE DONE.

*2.Please provide additional details regarding participant consent. In the ethics statement in the Methods and online submission information, please ensure that you have specified what type you obtained (for instance, written or verbal, and if verbal, how it was documented and witnessed). If your study included minors, state whether you obtained consent from parents or guardians. If the need for consent was waived by the ethics committee, please include this information.*

**Response:**Our research focuses on analyzing publicly published school textbooks, with no copyright disputes involved. It does not involve any human or animal subjects, nor does it involve collecting or harming any living organisms.

*3. Thank you for stating the following financial disclosure:*

*“Support from the key research program of Anqing Normal University “Research on Determining Primary School Chinese Language Teaching Content Based on Text Style（SK202208ZD）”, the National Education Science Planning Ministry of Education Youth Project “The Classification of Interdisciplinary Programs in Education in China and America: An Event History Model. (EIA 220526)”, and the project of Talent of Education Law in Shanghai (2023JYFXR077) is gratefully acknowledged.” Please state what role the funders took in the study. If the funders had no role, please state: "The funders had no role in study design, data collection and analysis, decision to publish, or preparation of the manuscript."*

**Response:** The funders had no role in study design, data collection and analysis, decision to publish, or preparation of the manuscript. We have further stated this in cover letter and manuscript.

4. *We note that your Data Availability Statement is currently as follows: All relevant data are within the manuscript and its Supporting Information files.*

*Please confirm at this time whether or not your submission contains all raw data required to replicate the results of your study. Authors must share the “minimal data set” for their submission. PLOS defines the minimal data set to consist of the data required to replicate all study findings reported in the article, as well as related metadata and methods (https://journals.plos.org/plosone/s/data-availability#loc-minimal-data-set-definition).*

*For example, authors should submit the following data:*

*- The values behind the means, standard deviations and other measures reported;*

*- The values used to build graphs;*

*- The points extracted from images for analysis.*

*Authors do not need to submit their entire data set if only a portion of the data was used in the reported study.*

*If your submission does not contain these data, please either upload them as Supporting Information files or deposit them to a stable, public repository and provide us with the relevant URLs, DOIs, or accession numbers. For a list of recommended repositories, please see https://journals.plos.org/plosone/s/recommended-repositories.*

*If there are ethical or legal restrictions on sharing a de-identified data set, please explain them in detail (e.g., data contain potentially sensitive information, data are owned by a third-party organization, etc.) and who has imposed them (e.g., an ethics committee). Please also provide contact information for a data access committee, ethics committee, or other institutional body to which data requests may be sent. If data are owned by a third party, please indicate how others may request data access.*

**Response:** All required data and raw data are provided in the text.

5. *PLOS ONE publication criteria require that the data presented in the manuscript must support the conclusions drawn, and submissions will be rejected if the interpretation of results is unjustified or inappropriate, so authors should avoid overstating their conclusions. We noted the following potentially overstated and/or unclear statements in the Results section that require revision:*

*Section 4.1.1:"This change signifies significant progress in gender equality"*

*It is unclear whether this statement is supported by the presented results, as the observed change appears to be small and statistical significance is not reported.*

**Response:** Thank you to the reviewers for their careful review and constructive criticism. We have changed this sentence to “This change signifies slight progress in gender equality”. See blue words in line 256. Thank you again to the reviewers.

*Section 4.1.3: "In contrast, the proportion of male characters has decreased to 48.58% in the 2023 edition, while the proportion of female characters has increased to 51.42% (see table 7), reflecting progress in gender equality."*

*We believe this may be an overstatement and would recommend rephrasing “reflecting progress in gender equality." to "which may reflect some progress in gender equality." or similar.*

**Response:** Thank you to the reviewers for their careful review and constructive criticism. We have changed this sentence to "which may reflect some progress in gender equality." see blue words line 324. Thank you again to the reviewers.

*Section 4.2.1: "Firstly, the identities of female writers are more diverse, such as journalists, marine biologists, playwrights, theorists, scientists, publishing house editors, modern architects, fantasy novelists, etc. among the newly added female writers"*

*This statement is unclear, as it is unknown what this diversity is being compared against. For example, comparison groups could include male writers of the same textbook editions or female writers of previous editions.*

**Response:** Thank you to the reviewers for their careful review and constructive criticism. We added detailed data and compared the identities of the female authors of the two versions of the textbook, see blue words from line 350 to line 357. Thank you again to the reviewers.

*Section 4.2.1: "They demonstrate the independent spirit of modern women, break through the constraints of traditional roles, show the potential for self-expression and realization, and contribute to social change and civilization development".*

*This statement does not appear to be supported by the data presented.*

**Response:** Thank you to the reviewers for their careful review and constructive criticism. This section is an explanation of the diversity of female authors in the new edition of the textbook. It may be overinterpreted. We have revised this section to read as follows, “In the new edition of the textbook, the identity of female authors is diversified, showing the breakthrough of contemporary women to traditional gender roles and their contributions to social civilization and progress”,see blue words from line 357 to line 360. Thank you again to the reviewers.

*Section 4.2.2 - "Regrettably, the 2023 edition of Chinese textbooks has omitted the text 'Face Changing' to circumvent the topic of gender equality, and no alternative texts with a similar theme have been provided".*

*This statement does not appear to be supported by citation, and more supported information regarding omitted text is needed.*

**Response:** Thank you to the reviewers for their careful review and constructive criticism. We have greatly expanded the quotation and discussion of the "face change" in the text, see blue words from line 408 to line 434. Thank you again to the reviewers.

*Finally, PLOS ONE requires that the language in submitted articles must be clear, correct, and unambiguous. In this case, the manuscript requires editing for English grammar and usage as well as for typographical errors.*

***Response:*** HAVE DONE.

Reviewer #1:

*This manuscript discusses the use of frequency, expression, illustrations, and other methods to enhance the potential gender inequality in middle school Chinese language textbooks. By elevating the image and perspective of women in the textbooks, it aims to raise awareness of gender equality among young people and achieve future gender equality in society.*

*At present, in the research of Chinese textbooks, there are already analytical literature on character images and roles in Chinese texts, with more emphasis on the educational impact of positive typical characters in texts on students' growth; In the study of character illustrations in textbooks, more attention is paid to whether illustrations affect students' concentration, while there is currently little research on the impact of gender on characters in Chinese textbooks.*

*Advantages: 1. This manuscript provides a sensitive perspective on the analysis of the number, roles, professions, and other aspects related to women in Chinese and other language textbooks. It shows that the frequency of women's appearance in textbooks is not high, which also reflects a global issue of gender discrimination against women in life, career, personal values, and other aspects. This perspective is very valuable.*

**Response:** Thank you to the reviewers for their positive comments on the value of this paper.

*Improvement: There are some areas that need improvement before the manuscript can be considered for publication.*

1. *Current achievements and research directions related to Chinese language textbooks.*

**Response:** Thank you to the reviewers for their careful review and constructive criticism. In section 2.1, we added references and analysis of the literature on gender issues in Chinese textbooks, see blue words from line 110 to line 116. Thank you again to the reviewers.

2. *Adding constructive suggestions on improving the social status of women in textbooks to the theoretical framework can enhance the comprehensiveness and developability of the article.*

**Response:** Thank you to the reviewers for their careful review and constructive criticism. In the last two paragraphs of the conclusion, we added discussions on strategies to address and improve gender bias in the textbook, see blue words from line 679 to line 691. Thank you again to the reviewers.

Reviewer #2:

*The article employs a well-structured mixed-methods approach, combining quantitative content analysis with qualitative critical discourse analysis. The quantitative analysis is particularly rigorous, offering a systematic breakdown of key indicators such as the frequency of male and female authors, characters, and protagonists. The inclusion of specific data points, such as the increase in female authors from 8.30% in 2001 to 9.09% in 2023, strengthens the credibility of the findings. Additionally, the qualitative analysis provides depth by uncovering implicit gender biases, such as the portrayal of Marie Curie alongside her husband rather than independently. This dual-method approach enhances the study’s comprehensiveness and allows for a nuanced understanding of gender representation trends.*

*The study is highly relevant within both local and global contexts. Gender representation in textbooks remains a significant issue worldwide, and the manuscript effectively situates its findings within broader international discourse by referencing studies from multiple regions. The focus on Chinese language textbooks, used by approximately 200 million students, fills a critical gap in the literature and offers insights that have direct implications for educational policy. The discussion on policy implications, particularly in light of China’s national commitment to gender equality, is particularly compelling. The study provides a balanced perspective, highlighting both progress and persistent challenges in gender representation over two decades.*

*The manuscript effectively identifies positive developments, such as the increasing number of female authors and the improved gender balance in family roles. However, it also underscores concerning trends, including the continued underrepresentation of women in social and professional spheres and the persistence of gender stereotypes. The discussion of regression in female protagonist representation, from 19.05% in 2001 to 13.11% in 2023, is especially noteworthy. The study’s critical engagement with these issues adds depth to the analysis and highlights the need for continued reform.*

**Response:** Thank you to the reviewers for their positive comments on the value of this paper.

*Despite these strengths, the methodology section requires further elaboration to enhance the study’s transparency and reproducibility. The manuscript does not provide sufficient detail on the coding process, inter-coder reliability, or statistical tests used in the quantitative analysis.*

**Response:** Thank you to the reviewers for their careful review and constructive criticism. To enhance the study’s transparency and reproducibility, We have revised the description of the encoding process,see “Quantitative study” and “Qualitative study” in paper. The coding process was independently calculated by the first author of the paper for 12 books for a long time, so errors are inevitable and it is impossible to estimate its inter-coder reliabiliby. We have stated this in the conclusion part, see blue words from line 674 to line 675. Since it is a full sample statistic for the textbook, there is no need for inferential statistics. Thank you again to the reviewers.

*Similarly, the description of the critical discourse analysis approach lacks specificity. The authors mention an “improved CDA,” but it is unclear what modifications were made and how they were implemented.*

**Response:** Thank you to the reviewers for their careful review and constructive criticism. To clarify the CDA method, we have explicitly cited three forms of improved CDA in our study, with a particular focus on Fairclough's dialectical-relational approach. We have provided an explanation of the specific features of each approach and highlighted their differences, see blue words from line 222 to line 233, from line 435 to line 474 and from from line 438 to line 526. Thank you again to the reviewers.

*The manuscript exhibits significant repetition, particularly in the Results and Discussion sections. Key points, such as the underrepresentation of women and the persistence of gender stereotypes, are reiterated multiple times without adding new insights.*

**Response:** Thank you to the reviewers for their careful review and constructive criticism. We deleted the verbose and repetitive parts of the conclusion and discussion to make the article more concise and clear. Thank you again to the reviewers.

*the underrepresentation of women and the persistence of gender stereotypes, are reiterated multiple times without adding new insights. Additionally, the discussion of regression in gender representation is presented in multiple sections but would benefit from a more integrated analysis.*

**Response:** Thank you to the reviewers for their careful review and constructive criticism. We have adjusted section “Underrepresentation of women: gender ideology lags behind the times” to make the analysis more comprehensive,see blue words in line 656. Thank you again to the reviewers.

*While the study references existing literature, the engagement with theoretical frameworks and global perspectives on gender representation in textbooks could be strengthened. The manuscript cites key scholars such as Sunderland and Beauvoir but does not fully incorporate theoretical discussions on how gender stereotypes influence students’ career aspirations and self-perception.*

**Response:** Thank you to the reviewers for their careful review and constructive criticism. We removed references to Sunderland et al in conclusion. Thank you again to the reviewers.

*Furthermore, drawing on global practices, such as UNESCO’s guidelines for gender-sensitive teaching materials, would provide a stronger foundation for the study’s policy recommendations.*

**Response:** Thank you to the reviewers for their careful review and constructive criticism. In the last paragraphs of the conclusion, We mentioned UNESCO's guidelines, see blue words in from line 686 to line 691. Thank you again to the reviewers.

*The manuscript’s readability is hindered by long, complex sentences and occasional grammatical errors. For example, sentences such as “The present study conducted a quantitative and qualitative analysis of numerous core indicators, and found that with the improvement of gender awareness in China, an increase in the representation of women in the recent edition has been reflected in several key dimensions” could be revised for conciseness and readability.*

**Response:** Thank you to the reviewers for their careful review and constructive criticism. We have corrected grammatical errors and long sentences in the text. Thank you again to the reviewers.

*The study would benefit from a more in-depth exploration of its practical implications for educational policy and practice. While the findings highlight gaps in gender representation, the manuscript does not provide concrete recommendations for textbook authors, policymakers, or educators. Additionally, the omission of texts addressing gender equality is noted, but the manuscript does not fully explore how this might influence students’ perceptions or what corrective actions could be taken.*

**Response:** Thank you to the reviewers for their careful review and constructive criticism. In the last two paragraphs of the conclusion, we added discussions on strategies to address and improve gender bias in the textbook, see blue words from line 679 to line 691. Thank you again to the reviewers.

*Finally, the manuscript would be strengthened by a discussion of counterarguments and study limitations. The analysis attributes gender disparities in textbooks primarily to editorial bias but does not consider alternative explanations, such as broader societal trends or historical constraints on available materials. Additionally, the study focuses on only two editions of Chinese textbooks, limiting its ability to capture long-term trends comprehensively. Acknowledging these limitations and discussing potential biases in the coding and analysis process would enhance the study’s credibility and scholarly contribution.*

**Response:** Thank you to the reviewers for their careful review and constructive criticism. This is indeed a limitation of the study, which we acknowledge in the second paragraph of the conclusion and on which we make recommendations for further research, see blue words from line 669 to line 675. Thank you again to the reviewers.

**Finally, We would like to express our sincere thanks to the editors and reviewers**
