# Supplementary material for: Progress and stagnation: A comparative study of gender representation in Chinese language textbooks for junior high school between 2001 edition and 2023 edition
Source: PLoS One. 2025 Nov 11;20(11):e0334398. doi: 10.1371/journal.pone.0334398 (PMC12604804; doi:10.1371/journal.pone.0334398)
Supplement: Attachment — Submitted filename: Response to Reviewers0807Y.docx [file pone.0334398.s002.docx]

Response to Editors and Reviewers

**Response to editors**

*We note that there were multiple versions of [Cover Letter, Manuscript, Revised Manuscript with Track Changes, Response to Reviewers] in your submission's file inventory. We have removed the older file(s), retaining the most recent version(s) for editorial review. Please double check your submission file inventory and let us know if any files appear to be outdated or absent.*

**Response:** We have checken our submission, and no files appear to be outdated or absent.

*Please provide additional details regarding participant consent. In the ethics statement in the Methods and online submission information, please ensure that you have specified what type you obtained (for instance, written or verbal, and if verbal, how it was documented and witnessed). If your study included minors, state whether you obtained consent from parents or guardians. If the need for consent was waived by the ethics committee, please include this information.*

**Response:** Our research focuses on analyzing publicly published school textbooks, with no copyright disputes involved. The images included in the Supplementary Information of this paper are sourced from publicly published textbooks, with no copyright disputes involved. The images include portraits of the text's author (e.g., S1 Fig., S2 Fig.), photographs of the main characters in the text (e.g., S3 Fig.), and illustrations of fictional characters from the novel (e.g., S4 Fig., S5 Fig.). None of the individuals depicted in the pictures are participants. The whole research does not involve any human or animal participant, nor does it involve collecting or harming any living organisms, and the Ethics Committee considered that an informed consent procedure was not required, therefore, we do not possess any authorization legal documents or ethical statements.

*Can you please upload an additional copy of your revised manuscript that does not contain any tracked changes or highlighting as your main article file.*

**Response:** We have done. I apologize for the oversight in the last version.

*Prior to sharing human research participant data, authors should consult with an ethics committee to ensure data are shared in accordance with participant consent and all applicable local laws.*

**Response:** The images included in the Supplementary Information of this paper are sourced from publicly published textbooks. These images include portraits of the author of the text (e.g., S1 Fig., S2 Fig.), photograph of the main characters in the text (e.g., S3.Fig), and illustrations of fictional characters in the novel (e.g., S4 Fig., S5 Fig.). These images are used exclusively for academic research purposes, in strict compliance with Article 24 of t*he Copyright Law of the People's Republic of China*, which permits the appropriate quotation of previously published works '*for the purpose of introducing, commenting on a certain work, or explaining a certain issue,*' provided that such use '*does not affect the normal exploitation of the work and does not unreasonably prejudice the legitimate interests of the copyright owner.*' Furthermore, such use '*may be undertaken without permission from the copyright owner and without payment of remuneration, provided that the author’s name (or pseudonym) and the title of the work are indicated.*'

Additionally, the use of portraits depicted in these images complies with Article 1,020 of t*he Civil Code of the People's Republic of China*, which stipulates that '*the use of a publicly disclosed portrait within necessary limits for personal study, artistic appreciation, classroom instruction, or scientific research*' '*may be made without the consent of the person whose portrait is used.*'

*Please provide additional details regarding participant consent.*

**Response:** Our research focuses on analyzing publicly published school textbooks, with no copyright disputes involved. It does not involve any human or animal subjects, nor does it involve collecting or harming any living organisms. Therefore, we do not possess any authorization legal documents or ethical statements.

*Please include a separate legend for each figure in your manuscript.*

**Response:** We have done. See blue words.

*Please upload a copy of Figure 1, 2, 3, 4 and 5 which you refer to in your text on page xx.*

**Response:** We have done. See blue words.

*Please review your reference list to ensure that it is complete and correct. If you have cited papers that have been retracted, please include the rationale for doing so in the manuscript text, or remove these references and replace them with relevant current references. Any changes to the reference list should be mentioned in the rebuttal letter that accompanies your revised manuscript. If you need to cite a retracted article, indicate the article’s retracted status in the References list and also include a citation and full reference for the retraction notice.*

**Response:**We removed these references:

“[42]Smith, S. L., and A. D. Granados. “Content Patterns and Effects Surrounding Sex-role Stereotyping on Television and Film.” In Media Effects: Advances in Theory and Research, edited by J. Bryant and M. B. Oliver, New York / London : Routledge; 2009. p.342–361.

[43] Midgley, C., DeBues-Stafford, G., Lockwood, P., & Thai, S. She needs to see it to be it: The importance of same-gender athletic role models. Sex Roles. 2021; 85(3): 142–160. DOI: 10.1007/s11199-020-01209-y”

These references were added:

“[19] Zhang, L., Zhang, Y., & Cao, R. "Can we stop cleaning the house and make some food, Mum?": A critical investigation of gender representation in China's English textbooks. Linguistics and Education. 2022; 69, 101058. https://doi.org/10.1016/j.linged.2022.101058

[37] Fairclough, N. Critical Discourse Analysis: The Critical Study of Language. Routledge. 1995, p. 133.

[39] Ueno, C. Patriarchy and Capitalism (Z. Yun & X. Mei, Trans.). Hangzhou: Zhejiang University Press. 2020, p. 48.”

Response to Reviewers

*Reviewer #2: Review of the article entitled “Progress and Stagnation: A Comparative Study of Gender Representation in Chinese Textbooks for Junior High School between 2001 Edition and 2023 Edition”*

*The authors have made commendable progress in response to reviewer feedback. The manuscript demonstrates increased clarity in its methodological description and improved engagement with international policy standards.*

**Response:** Thank you to the reviewer for his/her positive comments.

*However, further enhancement is still needed in terms of methodological transparency (e.g., coding examples), theoretical integration (particularly educational and philosophical perspectives), and critical discussion of structural influences and limitations. Addressing these remaining issues will significantly strengthen the rigor and scholarly contribution of the manuscript. Here I list my detailed comments:*

*1. Methodology*

*First-round comment:*

*“Despite these strengths, the methodology section requires further elaboration to enhance the study’s transparency and reproducibility. The manuscript does not provide sufficient detail on the coding process, inter-coder reliability, or statistical tests used in the quantitative analysis. Similarly, the description of the critical discourse analysis approach lacks specificity. The authors mention an ‘improved CDA,’ but it is unclear what modifications were made and how they were implemented.”*

*Authors’ response and assessment:*

*The authors have taken steps to clarify the coding criteria in Sections 3.2 and 3.3. For instance, in Section 3.2.1 (“Coding criteria”), they specify that “protagonists are the core characters who drive the plot, excluding background characters” (lines 156–158). In Section 3.3.2 (“Data analysis”), the framework for analyzing family roles has been elaborated, adopting a tripartite model of “economic contribution/emotional support/decision-making leadership” (lines 202–205). These additions improve the clarity of the coding scheme.*

*However, the revision still falls short in two areas. First, the absence of concrete coding examples (e.g., a sample coding table illustrating gender role labeling for a specific text) undermines methodological transparency and limits reproducibility.*

**Response:** Thank you to the reviewers for their careful review and constructive criticism. See the red words in Table 2 for details of revision. We have added coding cases to table 2 and providing necessary supplementary information.

*Second, the application of Critical Discourse Analysis (CDA) remains vague. While Fairclough’s three-dimensional framework is referenced, the operationalization—particularly how “power relations” are identified and coded—has not been sufficiently detailed.*

**Response:** Thank you to the reviewers for their careful review and constructive criticism. We use Fairclough's three-dimensional framework to carefully analyze some of the texts. See red words in the lines 314-319, and in the lines 489-606.

*2. Theoretical Engagement*

*First-round comment:*

*“While the study references existing literature, the engagement with theoretical frameworks and global perspectives on gender representation in textbooks could be strengthened. The manuscript cites key scholars such as Sunderland and Beauvoir but does not fully incorporate theoretical discussions on how gender stereotypes influence students’ career aspirations and self-perception. Furthermore, drawing on global practices, such as UNESCO’s guidelines for gender-sensitive teaching materials, would provide a stronger foundation for the study’s policy recommendations.”*

*Authors’ response and assessment:*

*The authors have incorporated UNESCO’s Gender-Sensitive Textbook Guidelines in the policy recommendations section (lines 686–691), advocating for “balancing gender roles and reducing stereotypical descriptions.” This is a welcome addition.*

*Nonetheless, one important gap remains. While the manuscript initially referenced Beauvoir, the revised version misses the opportunity to engage in a deeper philosophical dialogue—for example, by connecting the “othering” of female characters in textbooks with Beauvoir’s concept of women as a socially constructed “second nature.” These missed opportunities weaken the theoretical foundation of the study.*

**Response:** Thank you to the reviewers for their careful review and constructive criticism. We further explain the problem of "othering", see red words in the lines 730-737.

*3. Practical Implications*

*First-round comment:*

*“The study would benefit from a more in-depth exploration of its practical implications for educational policy and practice. While the findings highlight gaps in gender representation, the manuscript does not provide concrete recommendations for textbook authors, policymakers, or educators. Additionally, the omission of texts addressing gender equality is noted, but the manuscript does not fully explore how this might influence students’ perceptions or what corrective actions could be taken.”*

*Authors’ response and assessment:*

*The authors have addressed this point by offering specific suggestions in Chapter 6 (Conclusion). For instance, they recommend that textbook authors “design equal social roles, such as increasing the images of female scientists and engineers” (lines 679–680), and that policymakers “develop gender-sensitive teaching material guidelines and clarify gender equality assessment indicators” (lines 688–689). In Chapter 5 (Discussion), they also reflect on the removal of the text “Face-Changing,” arguing that it deprives students of the opportunity for critical engagement with gender issues (lines 408–434).*

*Despite these improvements, the influence of omitted gender-equality texts on students’ cognitive development is still not sufficiently addressed. Incorporating insights from educational psychology could help elucidate the link between textual omission and the reinforcement of gender stereotypes, thus enhancing the empirical and practical relevance of the study’s claims.*

**Response:** Thank you to the reviewers for their careful review and constructive criticism. We use educational psychology theory to further illustrate the impact of gender inequality on students' psychological development. See red words in the lines 31-33, and in the lines 704-709.

*4. Acknowledgment of Limitations and Counterarguments*

*First-round comment:*

*“Finally, the manuscript would be strengthened by a discussion of counterarguments and study limitations. The analysis attributes gender disparities in textbooks primarily to editorial bias but does not consider alternative explanations, such as broader societal trends or historical constraints on available materials. Additionally, the study focuses on only two editions of Chinese textbooks, limiting its ability to capture long-term trends comprehensively. Acknowledging these limitations and discussing potential biases in the coding and analysis process would enhance the study’s credibility and scholarly contribution.”*

*Authors’ response and assessment:*

*The authors now acknowledge in Chapter 6 (Conclusion) that analyzing only two editions restricts the longitudinal scope of the study. They recommend including additional editions in future research (lines 669–675). They also recognize the inherent subjectivity in the coding process and suggest adopting multi-coder strategies and reporting error indicators in future work.*

*However, a deeper engagement with alternative explanations is still missing. The manuscript briefly notes that “social and cultural traditions may have a greater impact on textbook content than editorial bias,” but this point is not fully developed.*

**Response:** Thank you to the reviewers for their careful review and constructive criticism.We have made an in-depth discussion of gender issues in Chinese culture, as shown the red words on lines 73-88,804-809, and 827-830.

**Finally, We would like to express our sincere thanks to the editors and reviewers**
