# Supplementary material for: Progress and stagnation: A comparative study of gender representation in Chinese language textbooks for junior high school between 2001 edition and 2023 edition
Source: PLoS One. 2025 Nov 11;20(11):e0334398. doi: 10.1371/journal.pone.0334398 (PMC12604804; doi:10.1371/journal.pone.0334398)
Supplement: Attachment — Submitted filename: Response to Reviewers914.docx [file pone.0334398.s003.docx]

Response to Editors and Reviewers

**Response to editors**

*1. If the reviewer comments include a recommendation to cite specific previously published works, please review and evaluate these publications to determine whether they are relevant and should be cited. There is no requirement to cite these works unless the editor has indicated otherwise.*

*2. Please review your reference list to ensure that it is complete and correct. If you have cited papers that have been retracted, please include the rationale for doing so in the manuscript text, or remove these references and replace them with relevant current references. Any changes to the reference list should be mentioned in the rebuttal letter that accompanies your revised manuscript. If you need to cite a retracted article, indicate the article’s retracted status in the References list and also include a citation and full reference for the retraction notice.*

**Response:** We checked our reference list, deleted two references and adjusted some references, as shown in blue words in reference list.

*PLOS ONE publication criteria require that the data presented in the manuscript must support the conclusions drawn, and submissions will be rejected if the interpretation of results is unjustified or inappropriate, so authors should avoid overstating their conclusions. We noted potentially overstated and/or unclear statements in the introduction and discussion sections that require revision. Please ensure all statements within the submission are appropriately supported by citations or the results presented in the manuscript.*

**Response:** Thank you to the editors for their careful review and constructive criticism. The paper has been carefully proofread and a lot of changes have been made to the problems mentioned by the editor. See the blue text in paper.

*Please ensure all statements within the submission are appropriately supported by citations or the results presented in the manuscript.*

*For example:Line 73-86: "China is an ancient country with the world-renowned Confucian culture. Confucianism has been traditionally valued in China to sustain a patriarchal society. Influenced by Confucian values, men are seen as representatives and inheritors of the family, while women are expected to marry into other families and continue their bloodline from the family they marry into. Therefore, men are encouraged to pursue careers, while women are required to take care of their husbands and children, look after their parents-in-law, and do laundry and cooking. The standard for measuring a woman's value is to be a "virtuous wife" to her husband's family. Housework is not recognized by society and lacks economic value. Men are authorities in the family domain, and their power is further strengthened and consolidated through gender division of labor in social life. This shows the "two-dimensional patriarchal system functioning in social and familial spheres" rooted in China [19]. Therefore, in ancient China, women were marginalized by society and their behavior and status were generally dominated by men"*

**Response:** Thank you to the editors for their careful review and constructive criticism，I've already deleted that part.

*"Since 2000, the status of Chinese women has seen tremendous improvement. In universities, the proportion of female students exceeds 50%, and in hospitals, the percentage of female doctors is over 46%. Furthermore, women account for 35% to 38% of corporate board directors and supervisors, and constitute more than 50% of grassroots neighborhood committee members."*

**Response:** Thank you to the editors for their careful review and constructive criticism, I've added two references to this section, see blue words in line 78-83.

*"Gogchild" (This is a typo, as it should be "Dogchild")*

**Response:** Thank you to the editors for their careful review and constructive criticism, we have corrected the error and therefore apologize.

*"however, there are few characters in Chinese textbooks that counter stereotypes."*

**Response:** Thank you to the editors for their careful review and constructive criticism，I've already deleted that part.

*"The patriarchal gender norm holds that women should be the other in the eyes of men and obey the will of men".*

**Response:** Thank you to the editors for their careful review and constructive criticism，I've already deleted that part.

**Response to Reviewers**

*Reviewer #2: Thank you for inviting me to review this manuscript for the third time. I truly appreciate your rigorous and thoughtful approach throughout this review process.*

*I have read the manuscript carefully and would also like to commend the authors for their careful revisions. The revised manuscript has adequately addressed all the concerns and suggestions I raised in my previous reviews. Therefore, I am pleased to endorse the publication of this article and would like to congratulate the authors in advance on its forthcoming acceptance and eventual publication.*

*Xiaoming*

**Response:** Thank you to the reviewer for his/her positive comments.

**Finally, We would like to express our sincere thanks to the editors and reviewers.**
